# Interacting impacts of hydrological changes and air temperature warming on lake temperatures highlight the potential for adaptive management

**DOI:** 10.1007/s13280-024-02015-6

**Published:** 2024-05-25

**Authors:** Freya Olsson, Eleanor B. Mackay, Bryan M. Spears, Philip Barker, Ian D. Jones

**Affiliations:** 1 UK Centre for Ecology & Hydrology, Bailrigg, Lancaster, UK; 2 Lancaster Environment Centre, Lancaster University, Bailrigg, Lancaster, UK; 3 Department of Biological Sciences, Virginia Tech, Blacksburg, VA USA; 4 UK Centre for Ecology & Hydrology, Bush Estate, Penicuik, Midlothian UK; 5 Biological and Environmental Sciences, University of Stirling, Stirling, UK

**Keywords:** Climate change, Climate mitigation, GOTM, Lake hydrodynamic modelling, Lake temperatures, River flow

## Abstract

Globally, climate warming is increasing air temperatures and changing river flows, but few studies have explicitly considered the consequences for lake temperatures of these dual effects, or the potential to manage lake inflows to mitigate climate warming impacts. Using a one-dimensional model, we tested the sensitivity of lake temperatures to the separate and interacting effects of changes in air temperature and inflow on a small, short-residence time (annual average ≈ 20 days), temperate lake. Reducing inflow by 70% increased summer lake surface temperatures 1.0–1.2 °C and water column stability by 11–19%, equivalent to the effect of 1.2 °C air temperature warming. Conversely, similar *increases* in inflow could result in lake summer cooling, sufficient to mitigate 0.75 °C air temperature rise, increasing to more than 1.1 °C if inflow temperature does not rise. We discuss how altering lake inflow volume and temperature could be added to the suite of adaptation measures for lakes.

**Supplementary Information:**

The online version contains supplementary material available at 10.1007/s13280-024-02015-6.

## Introduction

Lakes, globally, are exhibiting changes in thermal structure including higher water temperatures, longer and earlier periods of stratification, and loss of winter ice cover (Woolway et al. 2020). While these alterations are commonly linked to shifts in air temperature (AT; O’Reilly et al. 2015), various less-explored climate factors such as wind speed (Woolway et al. 2017), incoming solar radiation (Schmid and Köster 2016), and precipitation could also have significant impacts on water temperatures with subsequent implications for the onset and persistence of deepwater anoxia (e.g. Foley et al., 2012; Jane et al. 2021), metabolic rates (e.g. Kraemer et al., 2017), and other biogeochemical processes that are affected by water temperatures (Gibbons and Bridgeman 2020; Yin et al. 2023). The impact of these changes has not yet been well quantified but enumerating and understanding these impacts is crucial to apply effective management strategies to mitigate climate change. Furthermore, a thorough understanding of the impacts of these understudied drivers of lake change offers the prospect of widening the range of adaptive management interventions to mitigate climate impacts on lakes.

In small lakes, changes to river flows caused by changes in precipitation and management, such as abstraction and upstream-water storage, may be particularly relevant. Through-flowing rivers exert an advective heat flux on lakes (*Q*_adv;_ Livingstone and Imboden 1989; Schmid and Read 2021), and the effects of *Q*_adv_ are likely to be at their largest in small, short-residence time lakes, as the heat flux scales positively with discharge and inversely with lake size (Schmid and Read 2021). This flux has been shown to be a dominant process in some systems for determining lake water temperatures (Posada-Bedoya et al. 2021). Furthermore, recent modelling suggests that for lakes with an annual average residence time ≤ 100 days, inflow-outflow dynamics are required to simulate, accurately, lake temperatures (Almeida et al. 2022), and can also be important in lakes with longer water residence times (Valerio et al. 2015; Fenocchi et al. 2017).

Changes to river flows are driven by changes in precipitation and evapotranspiration, as well as abstraction pressures (Döll et al. 2009; IPCC 2014). These changes, predicted to impact more than 75% of the global landmass (van Vliet et al. 2013), can be very large, and, unlike air temperature warming, may be bi-directional. Projected changes to river flow range from large increases (> 50%) to large decreases (> 80%), depending on the climate projections, geographic region (van Vliet et al. 2013) and even among catchments in the same region (Fowler and Kilsby 2007; Garner et al. 2017). Additionally, predictions indicate increased seasonality in future river flows (van Vliet et al. 2013), with variability in both magnitude and timing of future flow changes. Despite the role of lake throughflows in the lake heat budget and the large variability in potential flow changes, few studies have considered the sensitivity of in-lake temperatures to a range of long-term shifts in river flows, choosing instead to focus on specific changes at the catchment scale (e.g. Bayer et al. 2013; Valerio et al. 2015) or responses to episodic changes such as under storm conditions (Anderson et al., 2020; Liu et al., 2020). The sensitivity of lakes to these long-term shifts is important in understanding how hydrological changes, driven by widespread climate change, will modify the functioning of lakes over timescales relevant for global processes.

Rivers have also experienced warming (e.g., Kaushal et al., 2010) and will continue to warm (van Vliet et al. 2013), although the effects of this river warming on lake water temperatures is not well known. As well as impacting river habitats, warming rivers will also affect lakes, by increasing the advective heat flux (Fink et al. 2014; Råman Vinnå et al. 2018). Quantifying the contribution of these interacting air temperature and flow effects (temperature and discharge) will provide a more complete analysis of the role of climate change on lake ecosystems and provide guidance on the potential for mitigation approaches.

Previous work in this area has generally focused on assessing the integrated impacts of climate change, where future changes are applied all at once (e.g. by using RCP scenarios from Global Climate Models) (Taner et al. 2011; Bayer et al. 2013; Valerio et al. 2015; Mi et al. 2020; Deng et al. 2023), often coupling these with catchment models to develop run-off and river flow scenarios that couple with lake models (Komatsu et al. 2007; Valerio et al. 2015; Deng et al. 2023; Jiménez-Navarro et al. 2023). These methods, although useful for understanding projected future in-lake conditions, present difficulties for isolating which changes have induced the observed response (e.g. Jiménez-Navarro et al. 2023) and therefore projecting how to utilise flow changes in management becomes challenging as the magnitude of the response to changes is unknown.

Despite the importance for smaller lakes outlined above, the impact of hydrological changes has often focused on large, deep systems (Posada-Bedoya et al. 2021; Deng et al. 2023), with some bias towards reservoirs (Guénand et al. 2020; Deng et al. 2023), which function differently to natural lakes (Hayes et al. 2017), especially in their hydro-morphology and inflow/outflow, both important drivers of lake temperatures (Kraemer et al. 2015). Small, natural, lake systems are an important type being both globally numerous (Messager et al. 2016) and disproportionately important for biogeochemical cycles (Harrison et al. 2009; Holgerson and Raymond 2016) and habitat resources (Scheffer et al. 2006; Downing 2010). The impact of inflow changes in these lake types has rarely been isolated from other climate impacts to quantify the response of in-lake temperatures to a large range of inflow conditions or to highlight the opportunity for adaptive management based on the potential for flow to mitigate air temperature warming.

Here, we use a one-dimensional hydrodynamic model to test the sensitivity of lake thermal conditions to incremental changes in river flow and air temperature. We assess the independent and interacting effects of plausible changes in air temperature and flow for a small, short-residence time lake in the northwest of England, highlighting the potential impact of air temperature warming and inflow for this lake type and the potential use of inflow management strategies to counter warming effects on lakes. Using ranges of future conditions predicted for this temperate region, we:

1. Assess how the individual and combined changes to river flow and air temperature impact water temperatures and water column stability.
2. Demonstrate the effects of inflow warming and quantify the contribution of this warming to the overall impacts.
3. Discuss how river interventions could be used to mitigate climate change impacts in lakes and inform adaptive management practices.

Though mentioned by previous studies (e.g. Bayer et al. 2013), we believe this to be the first study to examine how the sensitivity of lake temperatures to changes in inflows has the potential to mitigate climate warming impacts.

## Materials and methods

### Site description

A small, short-residence time lake in the northwest of England was selected as a case study to conduct the modelling. Elterwater (Lat: 54.4287, Long: − 3.0350) consists of three basins separated by narrow bars (see Olsson et al. 2022a). The modelling was conducted on the inner basin of the lake, Elterwater-IB (mean depth = 3.3 m, maximum depth = 6.5 m, surface area = 0.03 km^2^, annual water residence time = 10–20 days). Elterwater-IB is typically monomictic, usually stratifying continuously between March and October (see Olsson et al. 2022a). The basin is fed by one primary inflow to the northwest, plus several smaller ephemeral streams. The outflow of the basin to the east discharges into the middle basin of the lake via a narrow bar. The primary inflow is gauged, but water level records are not available.

### Hydrodynamic modelling

We used the lake version of the General Ocean Turbulence Model (GOTM version 5.4.0; see Burchard et al. 1999), a 1-D process-based physical lake model that uses measured meteorological data, inflow data, and bathymetry to model vertical mixing dynamics, lake heat fluxes, and water temperatures. Since its development, GOTM has been applied to a number of lakes, including Elterwater-IB (Olsson et al. 2022d), successfully replicating in-lake thermal conditions and mixing dynamics.

We configured the inflow using the density resolved method that intrudes the inflow to a depth of neutral buoyancy while also maintaining water balance, by assuming that outflow volume is equal to inflow volume. The outflow was configured as a surface outflow with a temperature equal to the surface water temperature. Assuming a water balance allows us to isolate throughflow effects separate from water level changes. Given these assumptions on the configuration of the inflows and outflows and the advective heat flux of throughflows (Eq. 1), we can assume that if the inflow temperature (*T*_in_) exceeds the outflow temperature (*T*_out_) then *Q*_adv_ will be positive (warming) and where outflow temperature (*T*_out_) exceeds the inflow temperature (*T*_in_) the flux will be negative (cooling). The heat flux of the throughflow (*Q*_adv_), assuming a stable water balance, is given by Livingstone and Imboden (1989) as,

1 $$Q_{{{\text{adv}}}} = \frac{{F_{{{\text{in}}}} \times C_{{{\text{pw}}}} \times \rho_{{\text{w}}} \times \left( {T_{{{\dot{\text{i}}\text{n}}}} - T_{{{\text{out}}}} } \right)}}{{A_{0} }}$$

where *F*_in_ is the discharge into the lake (m^3^ s^−1^), and (*T*_in_ − *T*_out_) is the temperature difference between the inflow and the lake outflow (°C), where the outflow temperature is assumed to be equal to the lake surface temperature. *A*_0_ is the lake’s surface area (m^2^) and *C*_pw_ and *ρ*_w_ are the specific heat capacity and density of water, given as the constants 4200 J kg^−1^ °C and 1000 kg m^−3^, respectively.

In this study, we used eight years (2012–2019) of driving data, modelling the system with 50 vertical layers at an hourly time step. Each year was run independently using the weather pattern for each individual year as the basis for climate forcing. This enabled evaluation of inter-annual differences in drivers and responses. From the eight separate years, we calculated the mean and standard deviation of in-lake effects.

#### Driving data

The required meteorological driving data (air temperature, wind speed, relative humidity, and short-wave radiation) were taken from an automated monitoring buoy at Blelham Tarn (Feuchtmayr et al. 2021, 2022). Blelham Tarn is close to the study site (< 5 km), is a similar size (0.1 km^2^), and has a similar elevation and fetch. The automated monitoring buoy records meteorological measurements at 4-min intervals, 2.5 m above the lake surface and an hourly mean was used as climate forcing. Inflow to Elterwater-IB from natural streams, ephemeral channels, and a pipeline constructed in 2016 to divert additional water into the lake was estimated for the modelling period (see Olsson et al. 2022a, d). Inflow temperature was measured at one inflow between July 2017 and December 2019. Outside of this period, inflow temperature was estimated using a linear relationship developed between the measured inflow temperature and the 12-h rolling average air temperature (see Supplementary Text 1 and Figure S1). Further details of the linear relationships and the gap filling protocol for meteorological, inflow discharge, and inflow temperature data can be found in Supplementary Text 2 and Olsson et al. (2022b, d).

#### Model calibration and validation

GOTM was calibrated for Elterwater-IB using hourly-average observations of lake temperature profiles from 2018 (Olsson et al. 2022c). Water temperatures were measured at 0.5, 1, 2, 3, 4, 5, and 6 m at the deepest point of the basin using RBR SoloT thermistors (RBR, Ottawa, Canada), accurate to ± 0.002 °C. Measurements were taken every 4 minutes and an hourly-averaged calculated that was compared with the modelled output during calibration.

We selected five model parameters to include in the calibration: three non-dimensional scaling factors relating to wind speed, short-wave radiation, and outgoing surface heat flux plus the physical parameters, minimum kinetic turbulence, and visible light attenuation. These parameters were calibrated using ParSAC (version 0.5.7; Bruggeman and Bolding, 2020), which maximises the log-likelihood using a differential evolution method. The calibration routine included 2000 simulations and was run three times to identify issues of equifinality. Each of the calibration runs produced similar parameter sets and so the mean of these was used as the final parameter values (Table 1). Using these final model parameters, root mean squared error (RMSE), mean absolute error (MAE), and Nash–Sutcliffe estimate (NSE) were calculated (Table 1). A model validation was carried out for 2019, using observations of water column temperatures. Model performance across all water column temperatures was good, irrespective of the metric used, in both the calibration (Figure S2; RMSE = 1.39 °C, MAE = 1.08, NSE = 0.92), and validation (Figure S2; RMSE = 1.42, MAE = 1.08, NSE = 0.85) periods as well as specifically for surface and bottom water temperatures and Schmidt stability (Table S2, Figure S3). See Supplementary Text 3 for the full procedure.

**Table 1:** ParSAC calibration and validation output and final parameter values. The parameters included in the automatic calibration procedure were non-dimensional scaling factors relating to short wave radiation (swr), surface heat flux (shf), and wind speed (wsf), minimum kinetic turbulence (k-min), and e-folding depth of visible light (g2)

Parameter	Minimum allowable value	Maximum allowable value	Final parameter value
swr	0.8	1.2	1.018
shf	0.8	1.2	0.801
wsf	0.8	1.2	1.2
k-min (m^2^ s^−2^)	1.4 e−7	1.0 e−5	1.4 e−7
g2 (m)	0.5	2.0	0.93

### Experimental scenarios

To assess the lake's sensitivity to changes in air temperature and flow, we chose to use an incremental ‘sensitivity’ approach in which plausible modifications to climatic variables are made (Abdo et al. 2009), based on the range of potential future changes to AT and flow within this temperate region (Supplementary Text 4). This method can be used to investigate a wide range of boundary conditions, test a system's sensitivity to stressors (Darko et al. 2019), and disentangle responses to co-occurring drivers (e.g. Gray et al. 2019; Soares and Calijuri, 2021). We chose this method as it allows us to investigate the underlying mechanisms and processes influenced by our target variables (Snortheim et al. 2017), including the contributions of atmospheric and flow-induced heating and cooling to water temperature dynamics, and thereby assess how adaptive management strategies could be employed.

Using the validated model, we ran 81 different scenarios. Firstly, nine temperature modifications were applied ranging from + 0 to + 4 °C of warming, in 0.5 °C increments, based on a potential extreme warming under highest emissions scenarios (IPCC 2014). Secondly, we applied nine flow increments to every timestep ranging from a 70% reduction to a 70% increase (− 70, − 50, − 30, − 10, 0, + 10, + 30, + 50, + 70%), based on previous catchment and hydrological modelling in the region, which showed that changes in seasonal river flow can be more than an order of magnitude higher than any annual changes: 10–80% reductions in summer and 0–40% increases in winter (Fowler and Kilsby 2007; Prudhomme et al. 2013; Supplementary Text 4). GOTM was then run for each combination of AT and flow changes (n = 81 scenarios). In these scenarios, the changes in AT were also applied to the inflow temperature, using the relationship developed between AT and water temperature observations (Supplementary Text 1).

In a second assessment, the same 81 scenarios were run, but without the AT changes applied to the inflow temperature, simulating conditions where inflow warming was mitigated. These two sets of scenarios, with and without inflow warming, allowed us to quantify the contribution of inflow warming independent of flow effects.

### Post-modelling analysis

Each of the 2 × 81 model scenarios were carried out for each of the eight years, independently, resulting in 1296 model runs. The impact of the flow and AT on surface and bottom water temperature (SWT, BWT) and Schmidt stability (*S*_T_), a measure of water column stability (Idso, 1973), calculated using the rLakeAnalyzer R package (Read et al. 2011), was assessed. SWT, BWT, and *S*_T_ were averaged at a seasonal timescale for each of the model runs and then the absolute and percentage difference from baseline conditions calculated. Baseline model runs were defined as those with no AT change and no inflow change (i.e., observed climate and hydrologic forcing). The seasons were defined as follows: spring = March, April, May; summer = June, July, August; autumn = September, October, November; winter = December, January, February.

Additional metrics were derived to investigate the mitigating and compounding impacts of flow changes. For each flow scenario, we estimated the equivalent AT change necessary to produce the same change in temperature or *S*_T_ (the ‘air temperature equivalent’), using linear interpolation of the AT incremental change scenarios (i.e., scenarios with no adjustment to flow). This metric places the impacts of flow changes into the context of AT warming. In addition, we calculated the ‘mitigation potential’ of river flow changes describing the amount of AT warming that could be offset by the cooling impact of changes in flow. We define the ‘mitigation potential’ as the cooling effect of a flow change relative to the baseline, as a proportion of the AT change. The mitigation potential quantifies the potential for flow changes to mitigate (or nullify) the impact of the AT change on water temperatures.

## Results

### How do the individual and combined changes to river flow and AT impact water temperatures and water column stability?

**Fig. 1:**
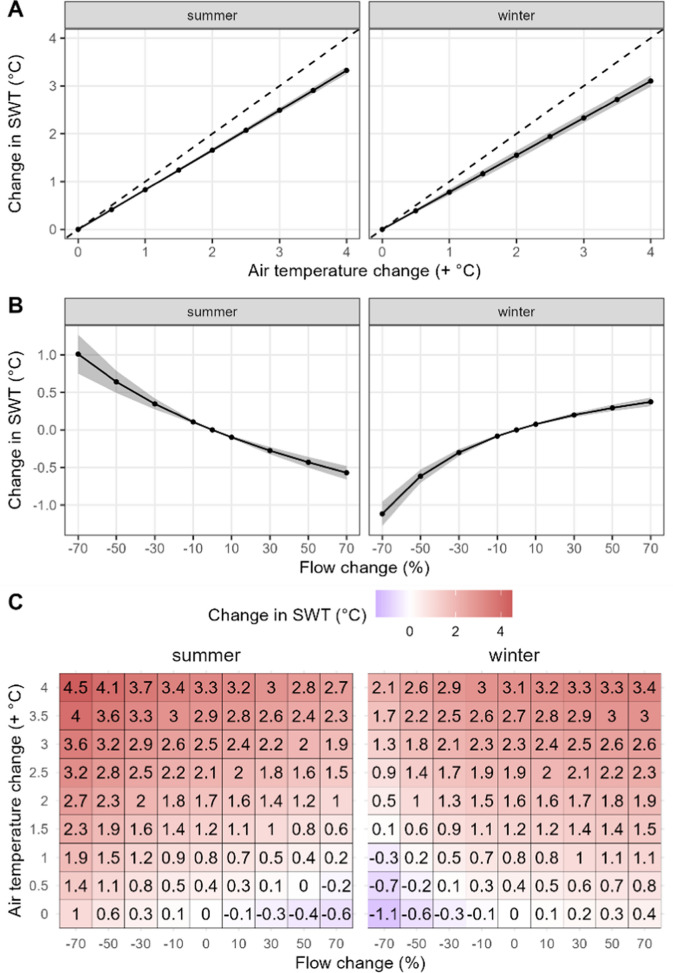
Change in summer and winter surface water temperature (SWT) compared to the baseline scenario (no flow or air temperature change). (**A**) change in SWT when only air temperature was changed (flow unchanged), (**B**) change in SWT when only flow was changed (air temperature unchanged), and (**C**) change in SWT under combined air temperature and flow changes. Grey shading on (**A**) and (**B**) show ± 1 standard deviation around the mean. Dashed line on panel (**A**) shows the 1:1 line

Under AT change only scenarios (air temperature warming + inflow warming, no flow change), lake surface water temperatures (SWTs) increased with AT warming. Mean SWTs increased linearly by approximately 0.4 °C for each 0.5 °C of AT warming, with the effect consistent between seasons (Fig. 1a, Figure S4).

In the flow-only change scenario, the direction and magnitude of SWT changes varied by season (Fig. 1b). In Elterwater-IB, lake inflow is generally cooler than surface lake water (and outflow) in the summer but warmer than lake water in the winter (Figure S5). Therefore, increases in flow cooled SWTs in summer but warmed SWTs in winter, with smaller changes to SWT (< 0.2 °C) in spring and autumn (Figure S4). Conversely, flow decreases caused warming of SWTs in the summer but cooling in the winter, of up to 1 °C warmer in summer and 1.1 °C cooler in winter at changes in flow of 70% (Fig. 1b).

The results for combined flow and AT changes showed that the impact of flow changes on summer SWT was relatively consistent irrespective of the magnitude of AT change. For example, 70% flow increases cooled summer SWT by − 0.5 to − 0.6 °C (difference between no flow change and a 70% increase in flow for each AT scenario, Fig. 1c). The difference in SWT changes between a no flow change and a 70% flow decreases was between 1.0 and 1.2 °C, depending on the rate of air temperature warming (Fig. 1c). For winter SWT, the cooling effect of a 70% flow reduction was 1.1–1.2 °C, while an increase of 70% in flow induced a warming of 0.3–0.4 °C, compared to the scenarios without flow change (Fig. 1c). The change in spring and autumn SWTs were smaller (Figure S4) and generally within one standard deviation of zero (no change). The potential variation in SWT impacts between scenarios with flow increases and flow decreases is substantial. When estimating SWT under different scenarios of rising ATs, there is a discrepancy in summer SWT of 1.6–1.8 °C between a scenario with a 70% increase and one with a 70% decrease in flow, while this discrepancy is 1.3–1.5 °C during winter (Fig. 1c).

BWT showed the same patterns to SWT but with a smaller magnitude of change (Figure S6). A seasonality in response to changes in inflow discharge was also observed where inflow increases caused cooling in summer and warming in winter, and inflow decreases caused warming in summer and cooling in winter (Figure S6b). Impacts of inflow decreases caused greater changes in BWT than inflow increases (Figure S6c) and were greater in summer and autumn than in spring and winter (Figure S6c).

**Fig. 2:**
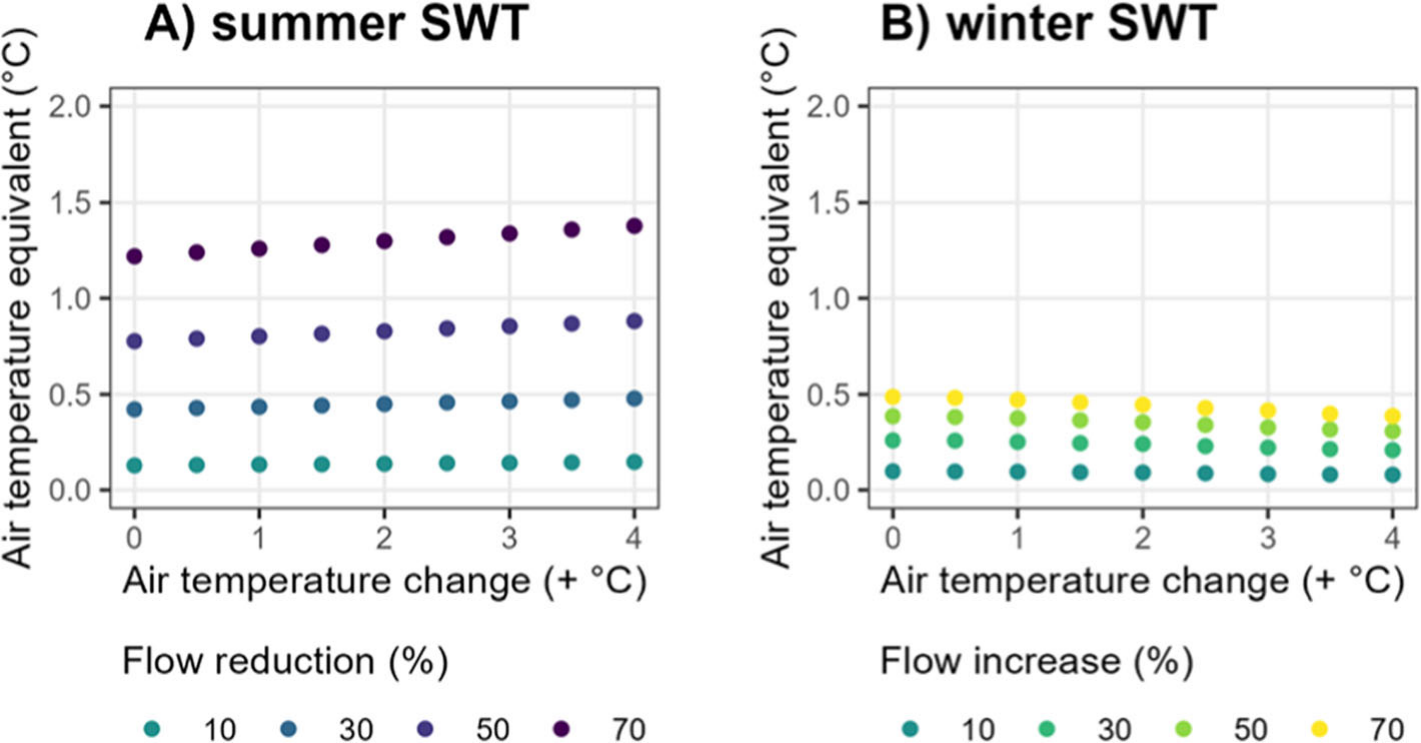
The air-temperature equivalent of the response of surface water temperature (SWT) to changes in flow in summer and winter. The x-axis denotes the air temperature addition to the baseline scenario. In summer, warming was induced by flow reductions and in winter, warming was induced by flow increases. Only flow scenarios that induced lake warming are included (reductions in summer and increases in winter)

The warming effects of flow changes on water temperatures can be compared to the AT change required to produce the same response (“air temperature equivalent”). The AT equivalent of a flow reduction of 70% for summer SWT was approximately 1.2 °C for the scenario with unmodified AT, increasing marginally with AT warming (1.4 °C when AT rose by 4 °C; Fig. 2a). Even smaller changes in flow (30%) had the equivalent impact of 0.4–0.5 °C additional AT warming on summer SWT (Fig. 2a). The SWT air temperature equivalents were overall lower in winter than in summer. However, the warming effect of increasing flow by 70% on winter SWT was still equivalent to a 0.4–0.5 °C AT rise, with the effect diminishing slightly at higher rates of AT warming (Fig. 2b).

**Fig. 3:**
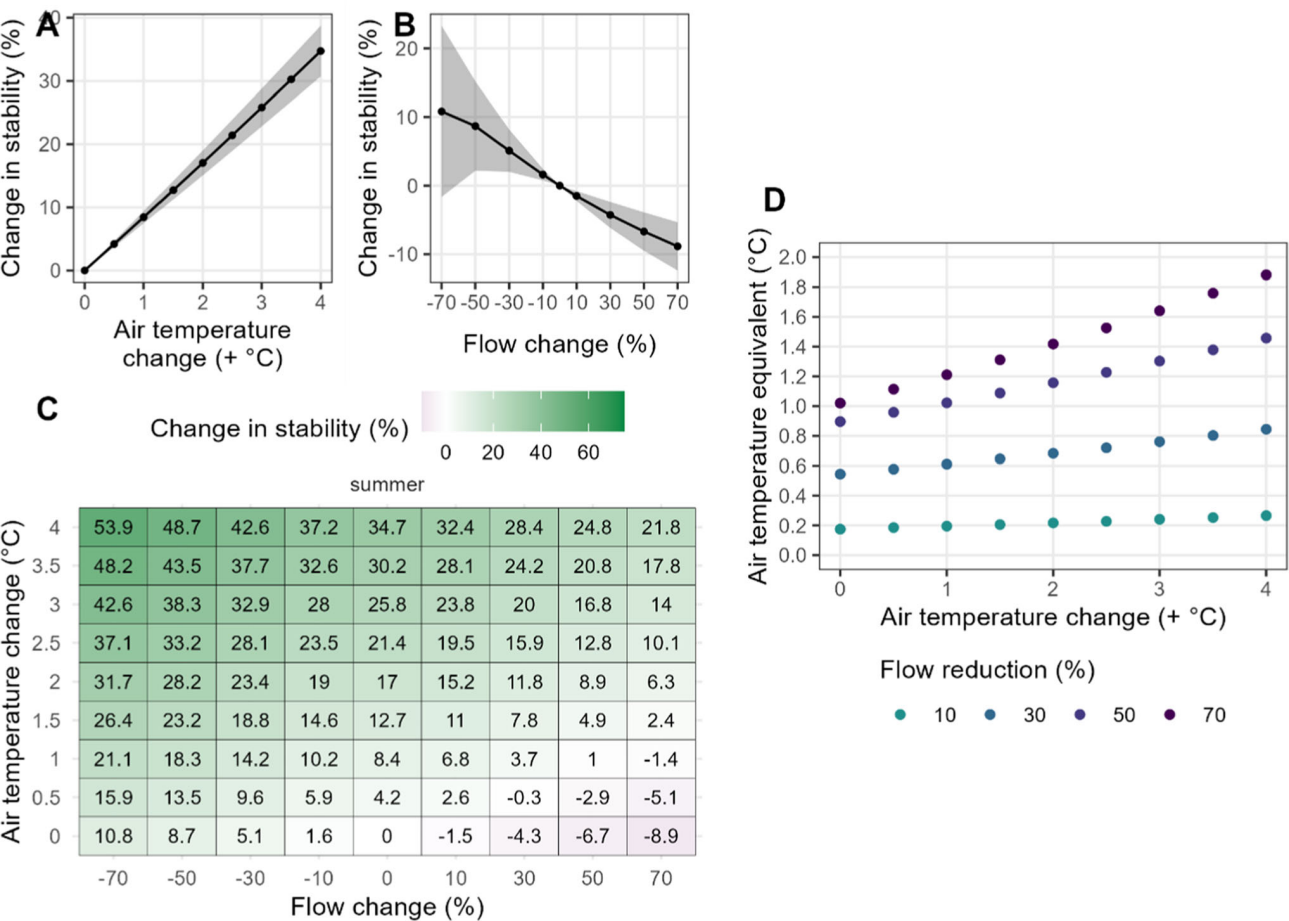
Percentage change in summer Schmidt stability (*S*_T_) compared to the baseline scenario (no flow or air temperature change). (**A**) change in *S*_T_ when only air temperature was changed (flow unchanged), (**B**) change in *S*_T_ when only flow was changed (air temperature unchanged), and (**C**) combined air temperature and flow changes. Grey shading on (**A**) and (**B**) show ± 1 standard deviation around the mean. (**D**) air-temperature equivalent of the response of summer *S*_T_ caused by flow reductions. The x-axis denotes the air temperature addition to the baseline scenario

Changes in AT and flow also had impacts on summer water column stability (as quantified using *S*_T_). Under warming AT and no flow change, the water column became more stable (Fig. 3a). Summer *S*_T_ increased linearly by 8 ± 0–4% per 0.5 °C of AT warming (Fig. 3a).

The cooling effect of the inflow in summer was reduced when flow was decreased, resulting in higher water column stability, although with high uncertainty (Fig. 3b). On average, a 70% reduction in flow resulted in a 11 ± 12.5% increase in *S*_T_ during summer (Fig. 3b). Conversely, *increased* flow reduced summer* S*_T_ due to the increased cooling effect of the inflow (Fig. 3b). Summer *S*_T_ was reduced by approximately 9 ± 3.5% when flow was increased by 70% (Fig. 3b).

The effect of the interacting impacts of decreased flow and increased AT on summer *S*_T_ were nonlinear, causing a greater increase in *S*_T_ than the addition of each change individually, especially at the highest rates of AT warming (Fig. 3c). For example, at 0.5 °C AT change, a 70% reduction resulted in an increase of 11.7% (15.9% increase compared to 4.2% with no flow change), whereas at 4 °C of AT warming, the same change in flow resulted in an increase in *S*_T_ of 19.2% (from 34.7% increase in *S*_T_ to 53.9%). The discrepancy between summer *S*_T_ under decreased flow scenarios (− 70%) and increased flow scenarios (+ 70%) at the highest temperature rise investigated here (4 °C), ranges from a 59% overestimation to a 55% underestimation of the percentage change in *S*_T_, if only AT changes are considered.

The response of summer *S*_T_ to the largest flow reduction tested (− 70%) was equivalent to the stability change caused by a 1.0–1.9 °C AT rise (Fig. 3d). These air-temperature equivalent effects increased at higher rates of AT warming given the interacting effects of AT warming and flow change. Smaller reductions in flow, of 30%, resulted in AT equivalent effects on summer water column stability of at least 0.5 °C for all AT warming scenarios tested (Fig. 3d).

### What is the effect and the contribution of inflow warming to the lake SWT changes?

#### Impact of inflow warming mitigation on AT effects

**Fig. 4:**
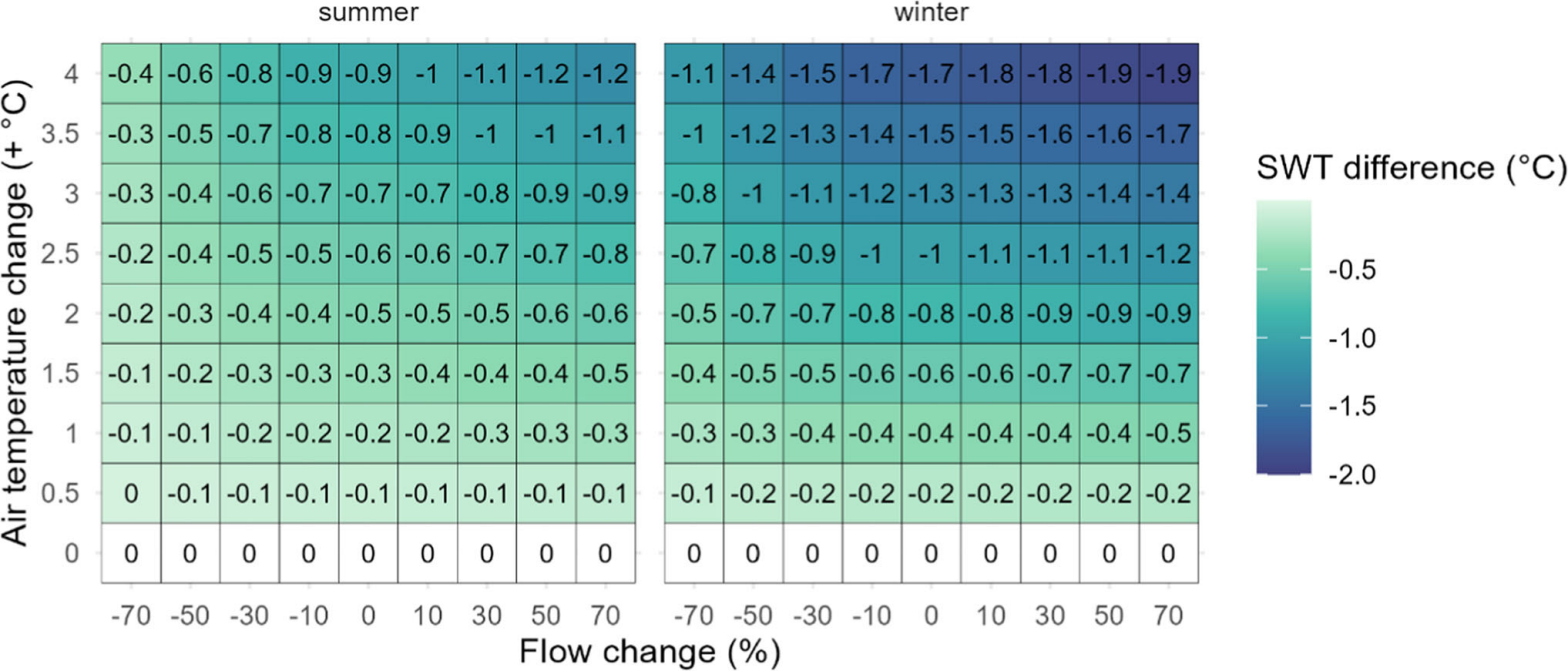
Difference in summer and winter surface water temperature (SWT) changes between scenarios with and without inflow warming mitigation. Negative values indicate that the scenario with inflow warming mitigation (no inflow warming) was cooler than the scenario with no inflow warming mitigation (inflow temperatures warmed)

In-lake impacts of the scenarios without inflow warming showed similar patterns to the scenarios with inflow warming (Figure S6). However, the effect of AT warming on SWT was less when the inflow was not warmed (Fig. 4), replicating a potential scenario where the inflow warming is mitigated. The values shown in Fig. 4 represent the difference in SWT changes with and without inflow warming mitigation. Looking at the AT effects in isolation of the flow change (flow change = 0), the lake SWT was cooler with inflow warming mitigation (no inflow warming), with the difference greater at higher rates of warming (Fig. 4, see Figure S7 for changes in SWT with inflow warming mitigation). At 4 °C of AT warming, the discrepancy was larger in winter than summer, with the SWTs up to 1.6–1.8 °C cooler with inflow warming mitigation (approximately 45–50% of the total impact of AT warming on SWTs; Fig. 4). In summer, when AT warmed by 4 °C, the SWT temperature difference with and without inflow warming mitigation was lower (0.9 °C cooler with), but still accounted for more than 70% of the total increase in SWT.

#### Impact of inflow warming mitigation on flow effects

Mitigating the inflow warming also impacted the effect of changing flow by increasing the cooling effect of flow in the summer, especially at higher AT changes (Fig. 4, Figure S6). For example, during a scenario with AT increasing by 4 °C and flow increasing by 70%, the summer SWT was 1.2 °C cooler when inflow warming was mitigated. Similarly, in-lake warming effects of reduced flow during summer were lower when inflow warming was mitigated (Figure S6), resulting in cooler water temperatures (Fig. 4).

### How could management utilise physical changes to lake inflow to mitigate in-lake climate change impacts?

We explore what could be achieved through artificial manipulation of the lake inflow (both flow and temperature), within the limits of those shifts expected by climate change (+/− 70% changes in flow and 0.5–4 °C AT warming). However, the timing of when and where to increase or decrease the flow are dependent on the heating or cooling effects observed. We report the *mitigation potential* as the air temperature change that could be offset by a change in flow, focusing on impacts on SWT in summer and winter and *S*_T_ in summer, as previous results showed that increases in flow mitigated AT warming in summer and decreases in flow mitigated AT warming in winter, given the inflow-lake temperature difference. Initially, this mitigation potential is reported for the scenario where inflow volume is altered in combination with management strategies that nullify any change in river temperature, such as by use of heat pumps or by shading, and then reported for the scenario where inflow volume alone is altered.

**Fig. 5:**
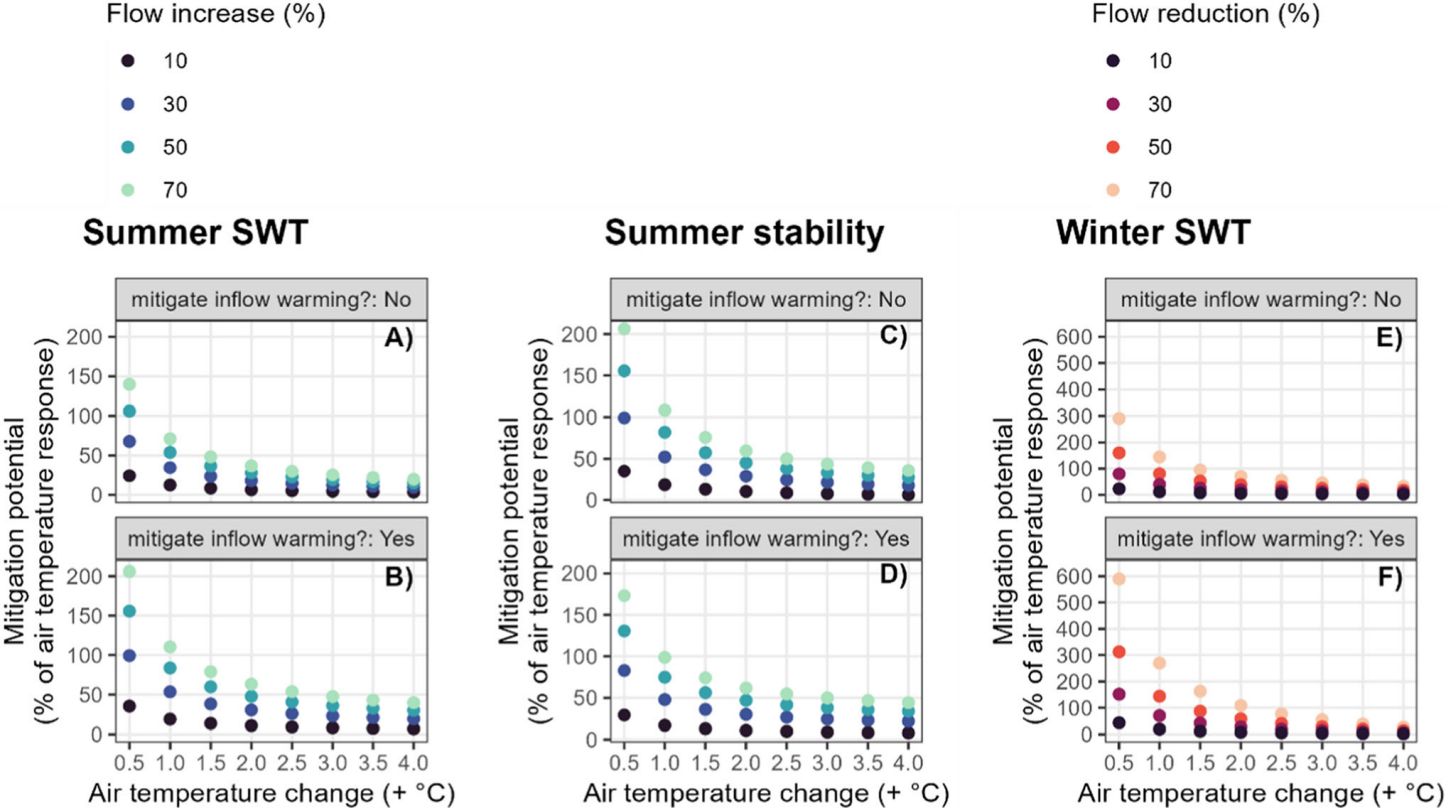
Potential of river flow changes to mitigate air temperature warming impacts on summer and winter surface water temperature (SWT) and summer water column stability. The mitigation potential is shown without (**A**, **C**, **E**) and with (**B**, **D**, **F**) inflow warming mitigation. Mitigation of in-lake impacts occurs in summer when flow is increased, and in winter when flow is decreased

The more that flow was increased in the summer the more it mitigated the impact of air temperature rise on water temperature and stability (Fig. 5). Conversely, the greater the change in air temperature, the greater its impact on water temperature and stability, and therefore a lower proportion of that change can be mitigated by a change in flow. Even relatively small increases in flow, combined with simultaneous mitigation of river temperature increases, could have a large impact when air temperature rises are modest, for example, a flow increase of 30% mitigated 100% of the effect of air temperature rising by 0.5 °C (Fig. 5b). While flow increases as much as 70% cannot completely mitigate the impacts of a change in air temperature of + 4 °C (Fig. 5a,c), when combined with mitigations of river warming, they still mitigated nearly 50% of the impact of the air temperature rise (Fig. 5b,d). The mitigation potential was even greater in the winter, when flow was decreased (Fig. 5f). When there was just a change in flow, with no management of river flow temperatures, the impacts were smaller (Fig. 5a, c, e), but still substantial.

## Discussion

These modelling experiments showed the importance of changes to flow for lake temperatures, and the potential for changes in flow to both exacerbate and mitigate AT warming. Predicted flow change for this region may lead to a water temperature increase equivalent to a 1.2 °C rise in AT, equivalent to multi-decadal trends in water temperatures (O’Reilly et al. 2015). In the present study, changes in SWT and *S*_T_ showed that the impact of flow was seasonal, driven by the relationship between the lake and inflow temperature. In our case study lake during summer, inflow temperature is lower than outflow temperature (assuming a surface outflow) causing cooling, whereas in the winter, inflow temperature exceeded outflow temperature, so the river flow warms the lake (Figure S5). In this region of northwestern Europe, projections under a moderate emissions scenario suggest that river flows, and therefore lake inflows, are likely to increase in winter (up to 70%) and decrease in summer (up to 75%; Fowler and Kilsby 2007; Prudhomme et al. 2013), potentially compounding AT warming impacts at both times of year.

### Potential for flow changes in management

Our results also showed that it was possible to cool lake water temperatures using flow modifications, relative to the no change scenarios, highlighting the potential for management of river flows to be used to mitigate climate-related impacts on lake water temperatures. Management actions, such as flow diversion (see Olsson et al. 2022a) or supplementation, which increases flows during times when the river is cooling the lake (summer in this case study), could mitigate a substantial part of the impacts of warming air temperature on lake temperatures and stability. This management approach could be applicable to a subset of lakes, particularly relatively small ones with nearby rivers where diversion of flow would be less engineering-restricted (Olsson et al. 2022a). However, novel management methods can provide “an extra tool in the toolbox” for managers and could be implemented in combination with other approaches. For example, we demonstrate that combining flow discharge changes with efforts to reduce the incoming water temperature can be more effective at mitigating in-lake warming. Furthermore, re-naturalisation of river flows and catchment flow processes (e.g. reducing summer abstraction in rivers; Döll et al. 2009) could have a positive benefit in mitigating warming in lakes, suggesting that management at a catchment level could have benefits not currently considered. Finally, flow changes of the magnitude discussed or even larger are projected from climate change (up to 80% reductions in summer flow; Prudhomme et al. 2013; van Vliet et al. 2013), requiring consideration in management decisions.

Cooling a lake will further impact other biogeochemical cycles. Warmer water temperature decreases solubility of dissolved gases (Jane et al. 2021), increase algal growth rates (Paerl et al. 2011), and warmer bottom water temperatures that can increase rates of internal loading (Gibbons and Bridgeman 2020; Yin et al. 2023). Limiting these processes could therefore improve water quality. Lakes with short-residence times (< 100 days) make up one quarter of lakes globally (Messager et al. 2016), suggesting a broad application of flow management for climate warming mitigation of lake temperatures.

### Considerations for climate change

Given the sensitivity of the lake temperatures to changes in inflow, management of lakes and reservoirs with short residence times must include an understanding of inflow impacts. Modelling studies which ignore changes in river flow under future climate, could be greatly under- or over-estimating future flow, with consequent errors in the lake temperature predictions, resulting in insufficient planning and inappropriate lake management practices being put in place.

Here, surface water temperature changes were underestimated by up to 1.2 °C when flow changes were not included at the highest rates of AT warming. This agrees with other modelling that showed epilimnetic temperatures were 3 °C too warm without the inclusion of inflows (Valerio et al. 2015), while another lake modelling study failed to accurately capture lake temperature dynamics without their inclusion (Almeida et al. 2022). Given around three quarters of the global land-surface is projected to be affected by river flow changes (van Vliet et al. 2013), the discrepancy between predictions with and without flow changes is globally relevant. Failing to include inflow warming would further underestimate climate related impacts of both AT warming and flow changes in relation to lakes (Valerio et al. 2015).

### Importance of inflow temperatures

The potential for the inflow to cause cooling (by increasing flow in summer, or decreasing flow in winter) was enhanced when inflow warming was mitigated (i.e., air temperature warming impacts on inflow temperature were not included). When inflow warming did not occur in the scenario, lake water temperatures were consistently cooler than when the inflow warming was not mitigated, by up to 1.5 °C at the highest rates of warming in winter (Fig. 5).

Given that up to 50% of the impact of AT warming came from the inflow temperatures increasing, management methods that prevent river warming may also mitigate against lake warming (see Orr et al. 2015). For example, riparian vegetation has been shown to reduce temperatures in rivers and streams (Ishikawa et al. 2021; Seyedhashemi et al. 2022). Therefore, planting riparian vegetation and forests (Turunen et al. 2021; Seyedhashemi et al. 2022) has the potential to limit warming in rivers and consequently, when upstream of lakes, limit lake warming or promote additional cooling depending on the current status of the inflow-lake temperature relationship. Riparian shading has been shown to be most effective at cooling streams in upland reaches (Orr et al. 2015; Seyedhashemi et al. 2022), where just 500 m of riparian shade could mitigate 1 °C of summer stream warming (Johnson and Wilby 2015). One coupled catchment-reservoir modelling study showed that riparian shading can have impacts on lentic inflow temperatures and reservoir inflow dynamics (Ishikawa et al. 2021), supporting our results for the potential for these measures to impact lakes. Another mitigation measure could be to utilise water heat pumps in rivers to generate renewable heating for buildings and businesses, whilst simultaneously reducing river water temperature (Gaudard et al. 2018).

In addition to changes in inflows modifying lake temperatures, as demonstrated here, and supported by other studies, these changes will also impact the hydrodynamics and mixing processes with further impacts on biogeochemistry. Consideration of the hydrodynamics of inflows, such as intrusions, plumes and flow pathways (Munar et al. 2018; Ishikawa et al. 2021; Posada-Bedoya et al. 2021) have demonstrated increases in underflows when inflows are cooler (Fenocchi et al. 2017; Ishikawa et al. 2021) and that inflows drive seasonal hydrodynamics in some systems (Munar et al. 2018; Posada-Bedoya et al. 2021), further demonstrating how long-term changes in inflow need to be considered in climate projections. These changes to mixing processes will be important for dynamics of nutrients and dissolved gases that are moved horizontally and vertically via these processes (Dresti et al. 2023; Gai et al. 2023).

### Limitations and further considerations

Our calibrated model captured the general dynamics of lake temperatures under baseline conditions, with an expected level of discrepancy from the observed conditions given the simplifications necessary when modelling (RMSE < 1.5 °C). Although not considered here in order to enable the isolation of inflow impacts, changes to inflows can also modify water level (Munar et al. 2018) with consequent impacts on lake temperatures (Rimmer et al., 2011; Munar et al. 2018). Similarly, the assumption of a surface outflow, with a temperature equal to lake surface temperatures, may also not be applicable in some systems (e.g. managed reservoirs with hypolimnetic withdrawals), again affecting the impact of flow changes (see Nürnberg, 2019). When considering adaptive management implementation at specific sites, rather than the sensitivity of variables, these uncertainties should be included in scenario-based simulations to fully capture the range of possible outcomes, and consideration given to the depth of any outflow and potential changes in water level. Given the potential water quality gains, but also the engineering required to modify flows, a thorough cost–benefit analysis should be used to elucidate the value of such management for specific systems.

## Conclusions

This work has demonstrated the sensitivity of lake temperatures to changes in flow for a small, short residence-time, temperate lake, a common lake-type globally. The impacts can be bidirectional, unlike AT warming impacts, and significant. Given the sensitivity of lake temperatures to inflow changes, adaptive management aimed at altering river flows and river temperatures could be an innovative approach to managing climate-related water quality issues related to rising water temperatures in lakes and be considered as one of a suite of the management tools available. The modelled impacts, however, were seasonal and dependent on the relationship between inflow and in-lake temperatures, requiring specific investigation at a regional or even for an individual lake scale to quantify specific future effects and appropriate management strategies. The potential size of the impact demonstrates the need for inflow dynamics to be understood and incorporated in future modelling and management plans for mitigating climate change impacts on lakes.

## Supplementary Information

Below is the link to the electronic supplementary material.

Supplementary file1 (PDF 2049 kb)

## Data Availability

GOTM configurations, modelling protocol, and post–modelling analysis code are archived on Zenodo (Olsson et al. 2024a) along with the archived model output (Olsson et al. 2024b). Raw meteorological and inflow data are published at Feuchtmayr et al. (2021, 2022), and Olsson et al. (2022b).
