# Supplementary material for: Interacting impacts of hydrological changes and air temperature warming on lake temperatures highlight the potential for adaptive management
Source: Ambio. 2024 May 25;54(3):402–15. doi: 10.1007/s13280-024-02015-6 (PMC11780241; doi:10.1007/s13280-024-02015-6)
Supplement: Supplementary file 1 — Supplementary file1 (PDF 2049 kb) [file 13280_2024_2015_MOESM1_ESM.pdf]

Ambio

Supplementary Information

This supplementary information has not been peer-reviewed.

Title: Interacting impacts of hydrological changes and air temperature warming on lake temperatures highlight the potential for adaptive management

Supplementary Text 1

Inflow temperature estimates

A relationship between the mean of the previous 12 hours air temperature and measured water temperature (Olsson et al., 2022) was developed using the measurements taken of air temperature and inflow temperature between July 2017 and Dec 2019. This relationship was used to estimate inflow temperature prior to these measurements (2012-June 2017) and under future air temperature rises. A linear regression with an intercept of 3.2 and a slope of 0.67 produced an R-squared of 0.880 and a RMSE of 1.30 °C (Figure S1). Assumptions of the model were checked and confirmed visually.

Olsson, F., Mackay, E. B., Jones, I. D., Barker, P., & Spears, B. M. (2022). Elterwater inner basin: Estimated inflow discharge and water temperature, 2012-2019. NERC Environmental Information Data Centre. https://doi.org/https://doi.org/10.5285/2883aaf1-6148-49cb-904a-d271a028c716

Supplementary Text 2

Gap filling protocol for meteorological data (2012-2019)

- Gaps of less than 24 hours were filled using linear interpolation, for solar radiation maximum gap = 6 hours

- For the gaps that remained we used comparisons between Blelham and Windermere buoy meteorological data and fitted linear models between the sites (Table S1). Windermere's automatic water quality monitoring buoy measures the same meteorological data as Blelham and is situated on the south basin, approximately 2 km to the E of Blelham

Table S1 Linear regression between Blelham and Windermere buoy data variables

Variable	% missing before	% missing after	Intercept	Slope	*p*	Adjusted r-squared
Air temperature	6.8	0	-0.978	1.056	< 0.001	0.957
Wind speed	4.4	0.3	0.090	0.548	< 0.001	0.572
Solar radiation	1.9	0	3.913	0.931	< 0.001	0.895
Relative humidity	4.2	1.3	-1.809	1.024	< 0.001	0.863
Surface water temperature	4.3	0.04	-0.639	1.047	< 0.001	0.924

For the remaining missing values the following protocol was used:

1. Wind speed – average wind speed 24 hours previous and 1 week after the timestep.

2. Relative humidity – linear regression with between Blelham and Esthwaite Water automated water quality monitoring buoy weather station data (intercept = -5.917, slope =1.07, $p < 0.001$, adjusted R-squared = 0.915).

3. Surface water temp – linear interpolation.

Solar radiation and cloud cover calculations

Calculations carried out using the R packages *suncalc* (Thieurmel & Elmarhraoui, 2019) and *insol* (Corripio, 2019)

1. The time steps before sunrise and after sunset (i.e. at night) were set to 0

2. We then calculated clear sky solar radiation. Clear sky solar radiation is dependent on the time of day and the day of the year.

3. The clear sky solar radiation (*max*) was compared with the observed solar radiation (obs) to estimate the cloud cover (between 0 and 1)

$$cloud\ cover = 1 - (\frac{obs}{max})$$

4. Night-time cloud cover was estimated as the mean cloud cover for the previous day

Thieurmel, B. & Elmarhraoui, A. (2019). suncalc: Compute Sun Position, Sunlight Phases, Moon Position and Lunar Phase. R package version 0.5.0. https://CRAN.R-project.org/package=suncalc

Corripio, J. G. (2019). insol: Solar Radiation. R package version 1.2.1. https://CRAN.R-project.org/package=insol

Supplementary Text 3

Calibration and validation procedure

The General Ocean Turbulence Model (GOTM) was calibrated for Elterwater-IB using observations of water temperature from 2018. An auto-calibration tool, ParSAC (Bruggeman & Bolding, 2020), was used for calibration. ParSAC estimates the best parameter set using a differential evolution method based on a maximum-likelihood measure. Each calibration routine had 2000 model runs, each with different parameter values, trending towards a best fit, based on the log-likelihood estimate. GOTM was calibrated using five parameters: three non-dimensional scaling factors relating to wind speed (wsf), short-wave radiation (swr), and outgoing surface heat flux (shf) plus the physical parameters minimum kinetic turbulence (k-min) and visible light attenuation (g2). Observations of Secchi disk extinction depth from 2018-2019 were used to give the range of visible light extinction (g2) in the calibration routine.

$$g_2 = \frac{1}{k}$$

where *k* the light extinction coefficient, derived from secchi disk extinction depths (Z_{SD}), calculated according to Kalff (2002):

$$k = \frac{1.7}{z_{SD}}$$

In addition, non-visible light attenuation was set to a constant value based on the median calculated in (Woolway et al., 2015).

We evaluated the outcome of the calibration protocol by looking at the error between modelled and observed water temperatures using three metrics: root mean square error (RMSE), Nash-Sutcliffe efficiency (NSE), and mean absolute error (MAE),

$$RMSE = \sqrt{\sum_{i=1}^{n} \frac{(mod - obs)^2}{n}}$$

$$NSE = 1 - \frac{\sum(obs - \mathrm{mod})^2}{\sum\left(obs - \overline{obs}\right)^2}$$

$$MAE = \frac{\sum|\mathrm{mod} - obs|}{n}$$

where *mod* and *obs* are the modelled and observed water temperatures.

The calibration routine was run three times to identify issues of equifinality and as none were observed, the mean of the parameter values calculated in each run was used. Using these estimated model parameters a validation was run using observations from 2019, with both calibration and validation periods showing good fit (Figure S2).

As Elterwater-IB is approximately 5 km away from Blelham Tarn to occur, parameters were tested within a realistic range to allow some scaling of atmospheric observations. A narrow range was adhered to, to ensure conditions remained realistic. The optimised parameters provided a reasonable fit to observed water temperatures. Potentially use of a wider range of parameters may have marginally improved model fits, but at the expense of being less realistic given the measured atmospheric conditions.

Model performance

The model performed well during the calibration, especially for the surface temperature and Schmidt stability (Table S2), with lower model performance for bottom water temperature. During the validation periods performance remained high, reducing slightly for surface water temperatures and stability and increasing for bottom water temperatures.

Table S2 Model performance during the calibration and *validation periods assessed using three metrics: root mean squared error (RMSE), Nash-Sutcliffe efficiency (NSE) and mean absolute error (MAE). Units for RMSE and MAE are in the native units for the variable of interest (water temperature = °C, Schmidt stability = J m^{-2}).*

	Water temperature						Schmidt stability	
	Surface		Bottom		All depths			
	Cal	Val	Cal	Val	Cal	Val	Cal	Val
RMSE	1.18	1.50	1.85	1.73	1.34	1.42	3.60	4.12
NSE	0.96	0.89	0.27	0.36	0.92	0.85	0.90	0.60
MAE	0.87	1.05	1.39	1.28	1.08	1.09	2.05	2.49

Woolway, R., Jones, I., Feuchtmayr, H., & Maberly, S. (2015). A comparison of the diel variability in epilimnetic temperature for five lakes in the English Lake District. Inland Waters, 5(2), 139–154. https://doi.org/10.5268/IW-5.2.748

Supplementary Text 4

Future river flows under climate change

Future river flow for the Brathay catchment, in which Elterwater is situated, is predicted in the Future Flows dataset (Prudhomme et al., 2013). Future flow predictions show uncertainty based on the ensemble of 11 future predictions. The direction and magnitude of the predictions are variable – especially in spring and autumn where the direction of change is more uncertain. Spring ranges from +30 to –13% change, although the central estimates (median and mean) are small (around <1% reductions). Autumn similarly shows a +21 to – 25% change, although the central estimates are also small reductions (2-4%). Changes in summer are more consistently for a decrease (median -30%) and in winter an increase (median +15%). The mean change for the whole period is small compared to the potential changes in each season. The reductions in summer and largely compensated for by increases in winter flow.

Table S3 Seasonal summary of future flow changes (%) in the Brathay catchment from Prudhomme et al. (2013) under A1B emissions scenario (based on UKCP09 modelling).

**Season**	**maximum**	**minimum**	**median**	**mean**
**winter**	35	-3	15	14
**spring**	30	-13	-0.6	0.4
**summer**	6	-61	-30	-25
**autumn**	21	-25	-4	-2
**Annual**	9	-7	0.3	0.2

Table S4 Monthly summary of future flow changes in the Brathay catchment (%) from Prudhomme et al. (2013) under a A1B emissions scenario (based on UKCP09 modelling).

Month	Maximum	Minimum	range	median	mean
**Jan**	50	-17	67	17	18
**Feb**	40	-15	55	13	15
**Mar**	69	-24	93	-6	0.3
**Apr**	35	-32	67	8	3
**May**	54	-22	76	-4	2
**Jun**	42	-55	97	-17	-9
**Jul**	33	-55	88	-31	-19
**Aug**	8	-74	82	-34	-37
**Sep**	1	-60	61	-30	-26
**Oct**	24	-26	50	5	0.1
**Nov**	57	-2	59	3	12
**Dec**	39	-8	47	11	11

Monthly changes generally show the pattern observed in the seasonal data (reduction in Jun/Jul/Aug/Sep, increases in Nov/Dec/Jan/Feb). Estimates for June predictions range from a 53% increase to a 61% increase but all months show a large possible range of changes – highlighting the importance of looking at a range possibilities in the study.

Supplementary Figure 1

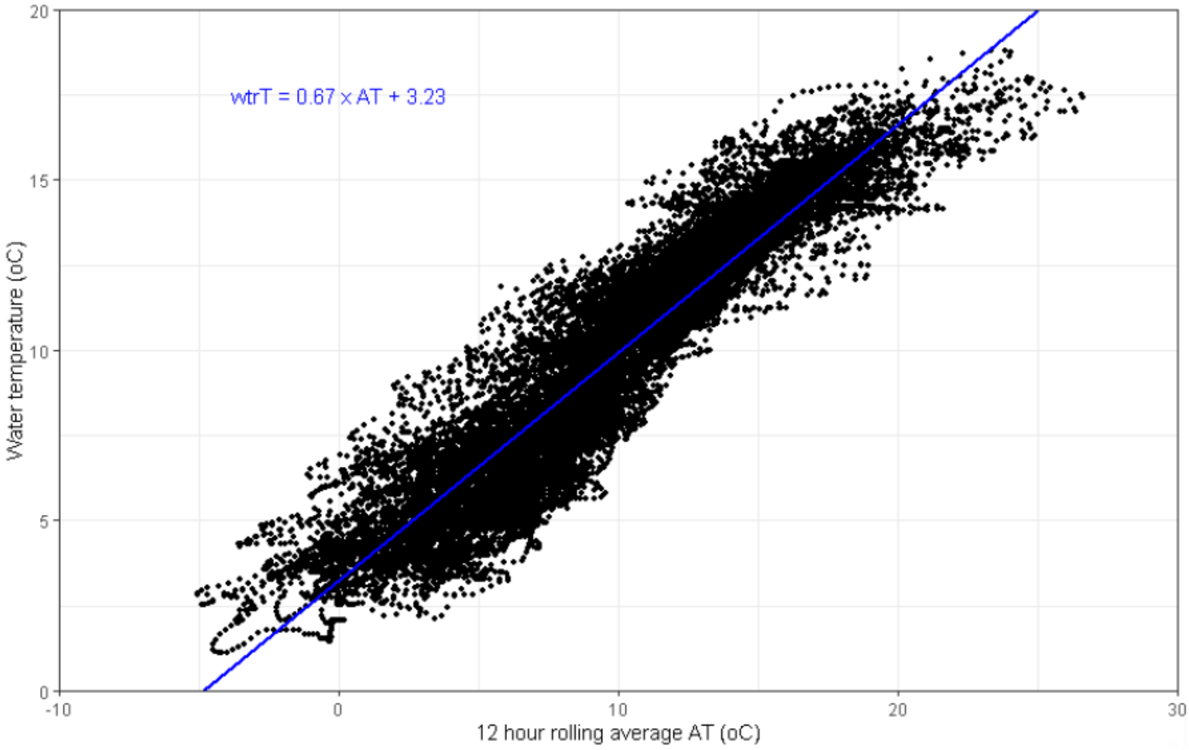

Figure S1 Prediction of Elterwater-IB (inner basin) inflow water temperature (Lat: 54.4287, Long: -3.0350) based on the 12-hour rolling average air temperature (AT) measured on the weather station at Blelham Tarn (Lat: 54.3959 Long: −2.9780). Points show observations and blue line the fitted linear model between the inflow temperature and air temperature.

Supplementary Figure 2

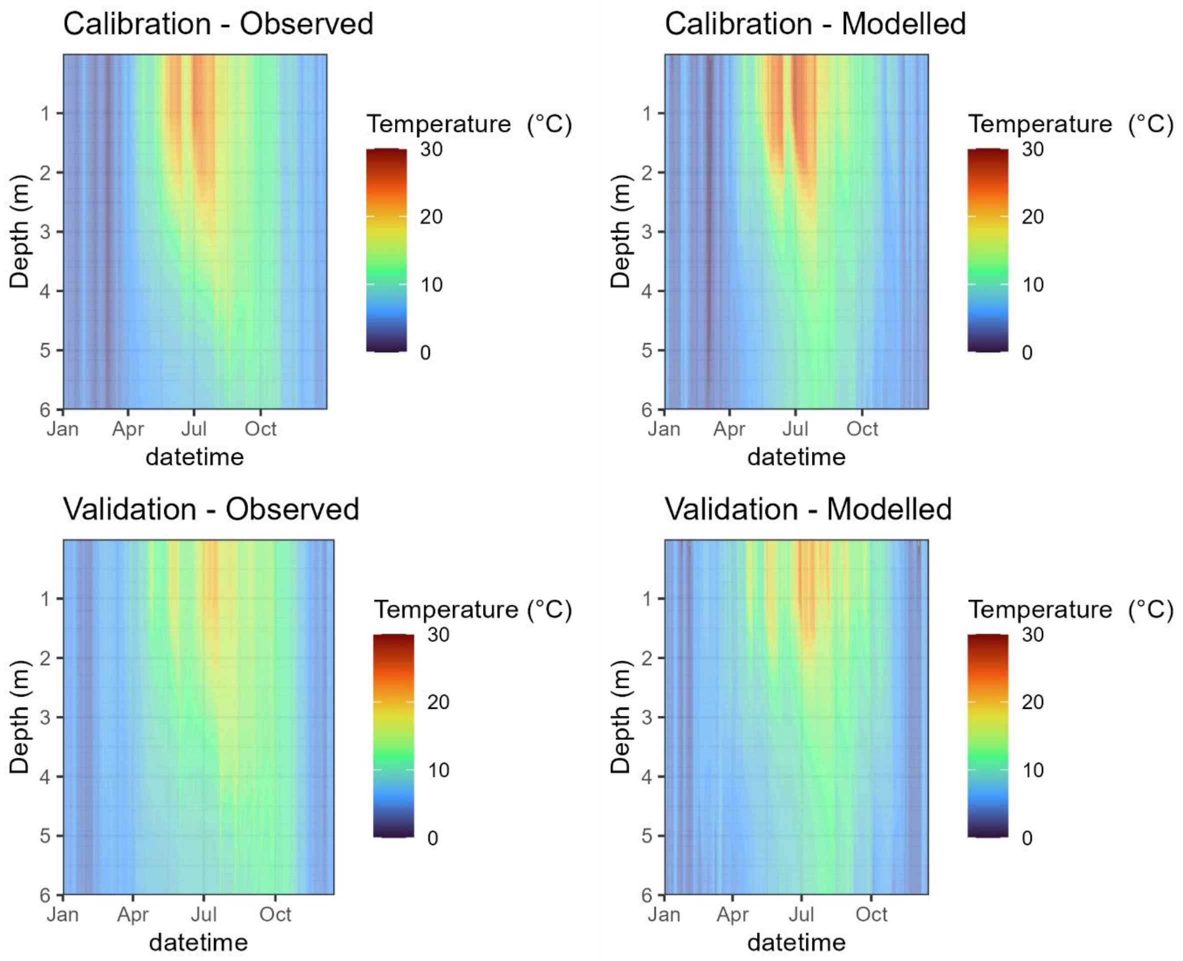

Figure S2 Model fit between observed and modelled water temperatures for the calibration period (2018) and validation period (2019).

Supplementary Figure 3

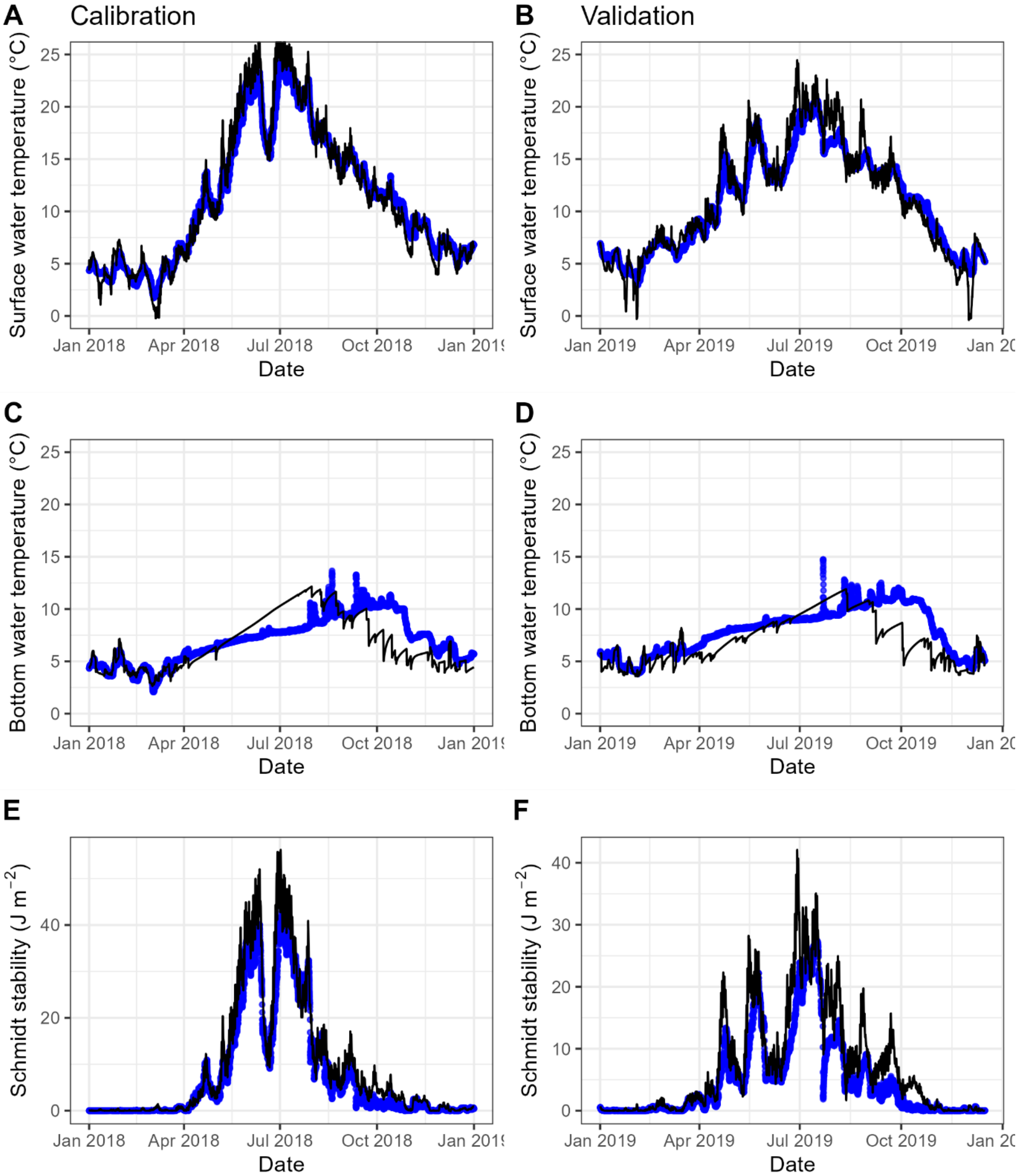

Figure S3 Model fit between observed and modelled water temperatures (A, B) and schmidt stability (C,D) for the calibration (2018) and validation periods (2019). Observations are shown by the blue points and model output by the black line.

The during the calibration and validation periods the root mean squared error for Schmidt period was 3.6 and 4.1 J m^{-2}, the Nash Sutcliffe Efficiency was 0.90 and 0.60, and the mean absolute error was 2.1 and 2.5 J m^{-2}.

Supplementary Figure 4

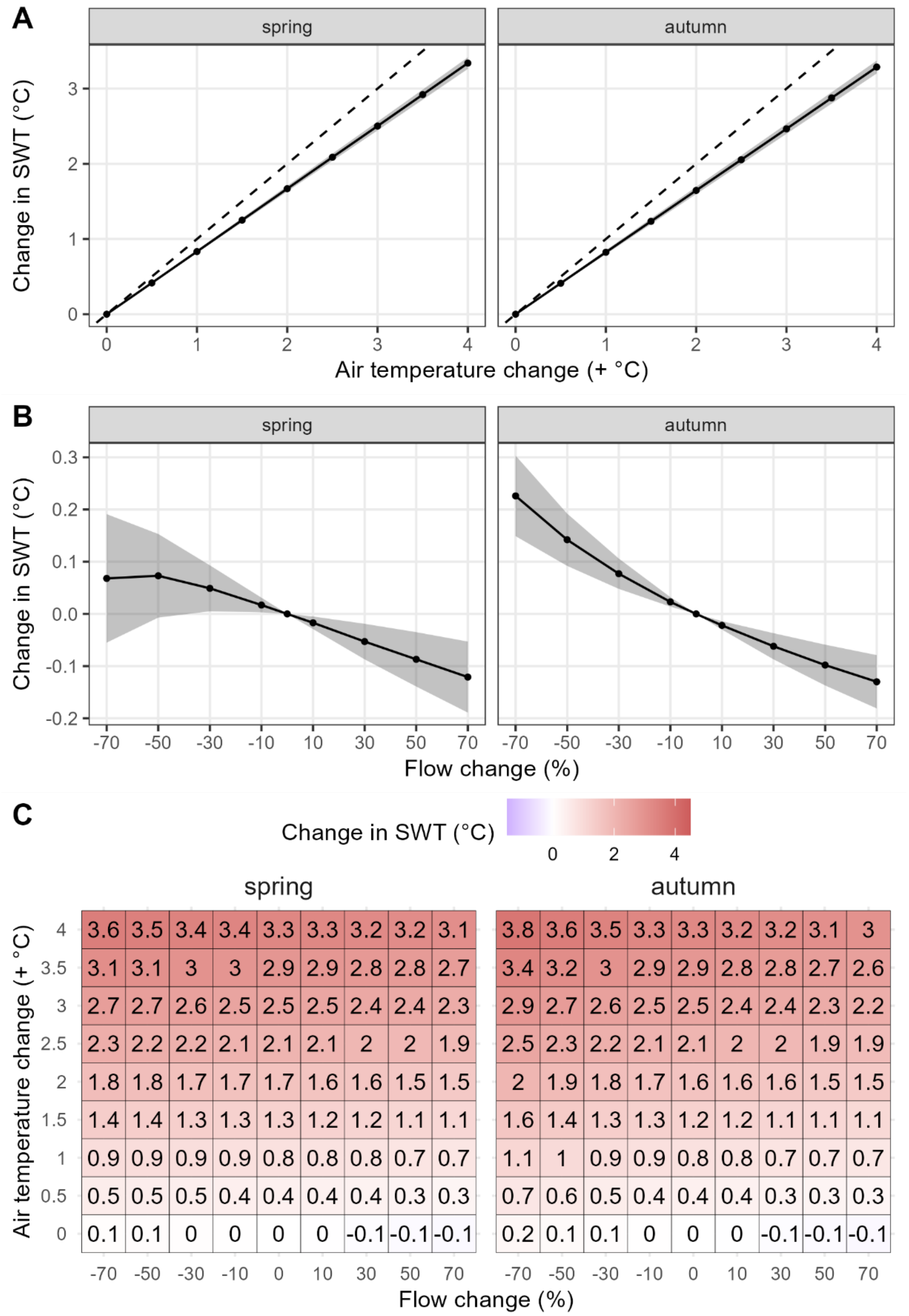

Figure S4 Change in spring and autumn surface water temperature (SWT) for A) air temperature change only (flow unchanged), B) flow changes only (air temperature unchanged), and C) combined air temperature and flow changes. Values represent the difference from the baseline scenario (unmodified air temperature and flow conditions). Grey shading on A) and B) show ± 1 standard deviation around the mean. Dashed line shows the 1:1 line.

Supplementary Figure 5

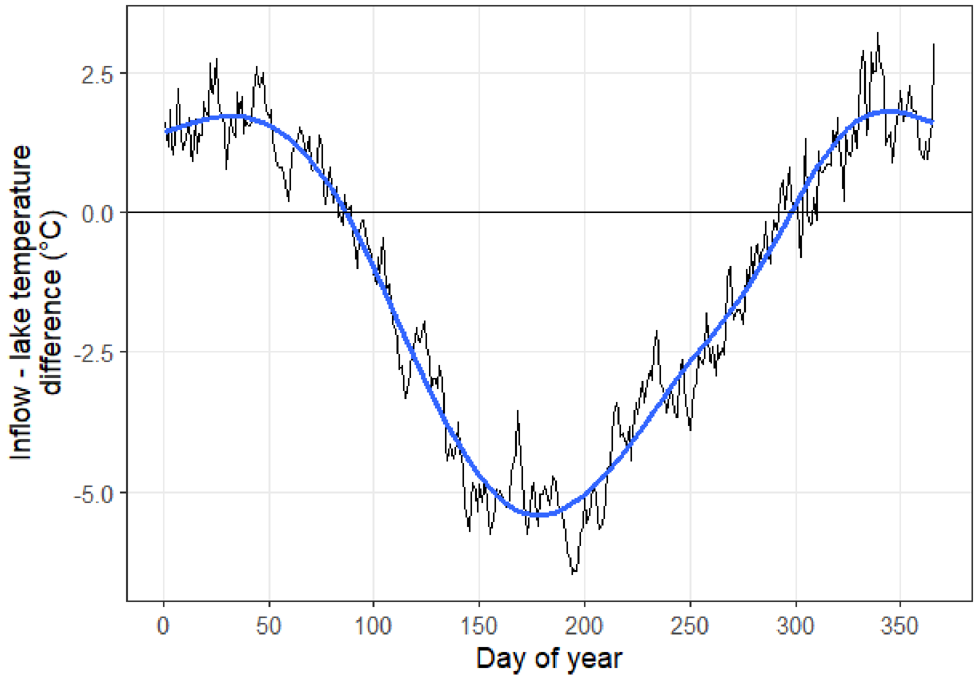

Figure S5 Inflow-lake temperature difference. Positive values are where the inflow temperature is greater than the lake surface temperature, and negative values are where the inflow temperature is less than the lake surface temperature. Data shown are averages values for each day of the year using 2012-2019 data. Blue line is a fitted general additive model to highlight the general pattern.

Supplementary Figure 6

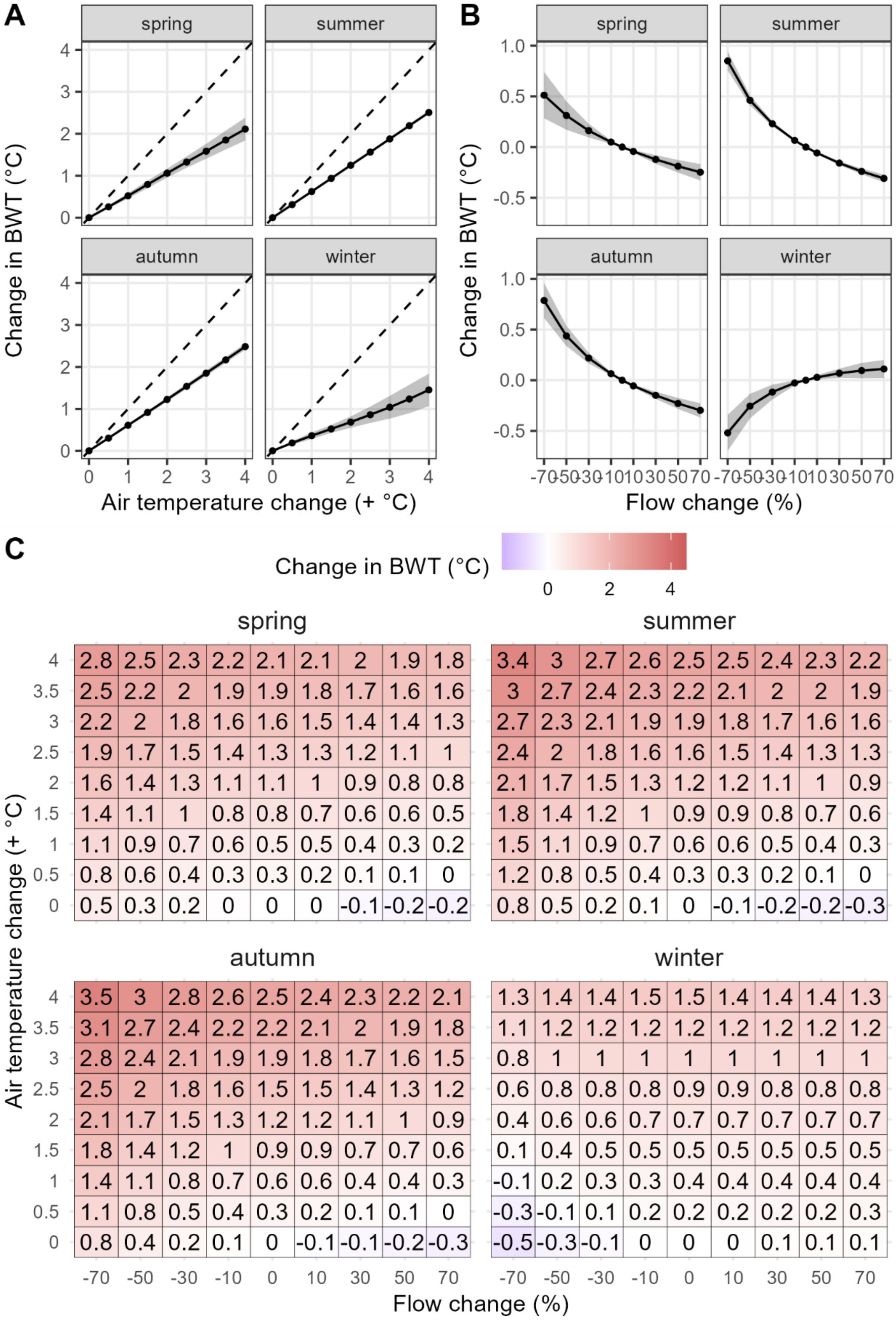

Figure S6 Change in bottom water temperature (BWT) for A) air temperature change only (flow unchanged), B) flow changes only (air temperature unchanged), and C) combined air temperature and flow changes. Values represent the difference from the baseline scenario (unmodified air temperature and flow conditions). Grey shading on A) and B) show ± 1 standard deviation around the mean. Dashed line shows the 1:1 line.

Supplementary Figure 7

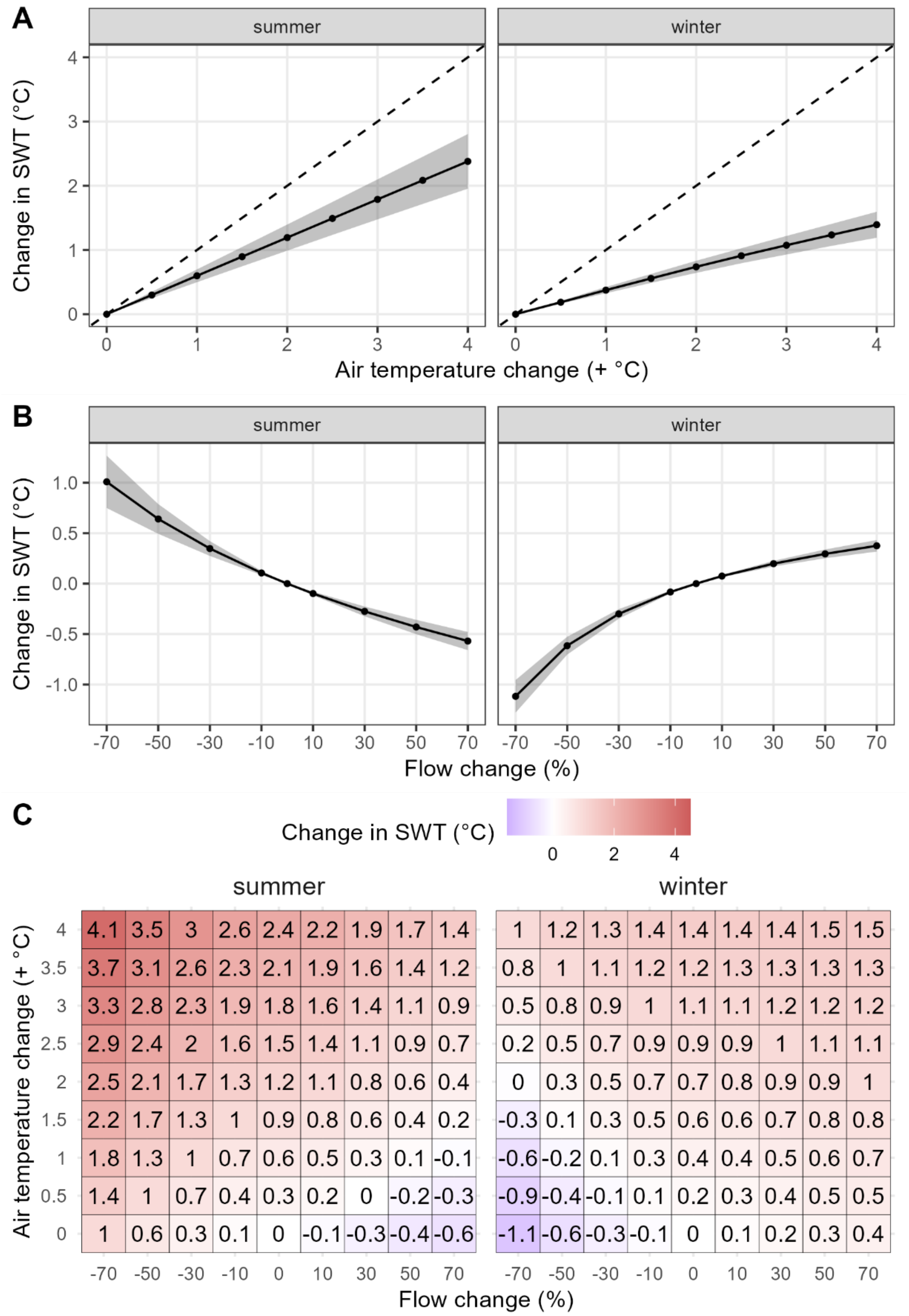

Figure S7 Change in summer and winter surface water temperature (SWT) when inflow warming mitigation is applied (i.e. no inflow warming) for A) air temperature change only (flow unchanged), B) flow changes only (air temperature unchanged), and C) combined air temperature and flow changes. Values represent the difference from the baseline scenario (unmodified air temperature and flow conditions). Grey shading on A) and B) show ± 1 standard deviation around the mean. Dashed line shows the 1:1 line.
